# Supplementary material for: Predicting visual function by interpreting a neuronal wiring diagram
Source: Nature. 2024 Oct 2;634(8032):113–23. doi: 10.1038/s41586-024-07953-5 (PMC11446822; doi:10.1038/s41586-024-07953-5)
Supplement: Supplementary file 6 — CRF and ERF predictions for individual TmY4 and TmY9 cells. Analogous to Supplementary Data 3, but for TmY target types. Shown are the top four monosynaptic pathways, the strongest pathway passing through each of the top ten intermediary types (ranking from Extended Data Fig. 7), and the trisynaptic pathway Tm1–TmY–Dm3–TmY (see the section entitled Prediction of spatial normalization). [file 41586_2024_7953_MOESM6_ESM.zip › DataS4/TmY9qΓèÑ/720575940648469881.pdf]

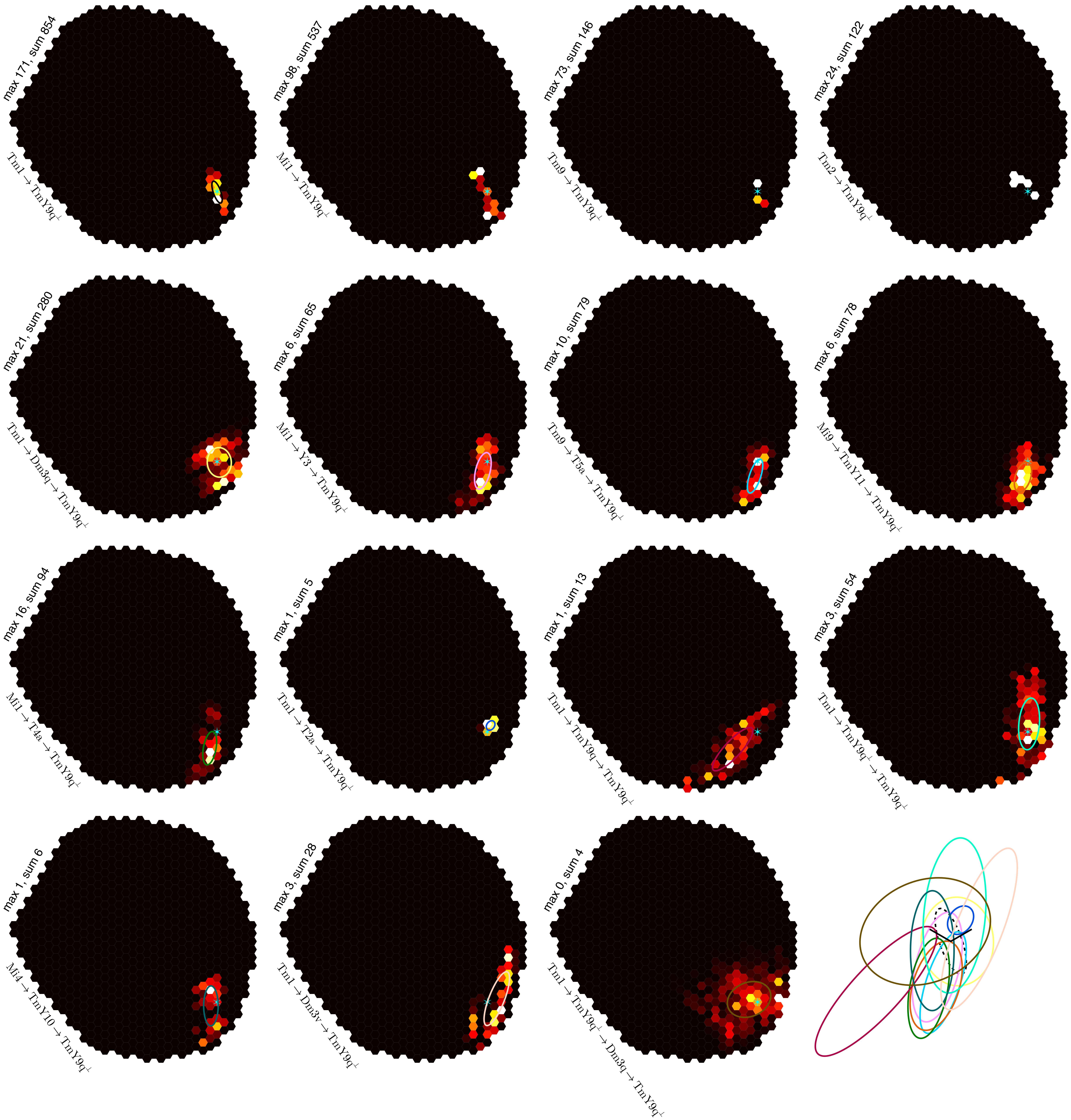

max 171, sum 854
Tm1 → TmY9q⊥
max 98, sum 537
Mi1 → TmY9q⊥
max 73, sum 146
Tm9 → TmY9q⊥
max 24, sum 122
Tm2 → TmY9q⊥
max 21, sum 280
Tm1 → Dm3q → TmY9q⊥
max 6, sum 65
Mi1 → Y3 → TmY9q⊥
max 10, sum 79
Tm9 → T5a → TmY9q⊥
max 6, sum 78
Mi9 → TmY11 → TmY9q⊥
max 16, sum 94
Mi1 → T4a → TmY9q⊥
max 1, sum 5
Tm1 → T2a → TmY9q⊥
max 1, sum 13
Tm1 → TmY9q⊥ → TmY9q⊥
max 3, sum 54
Tm1 → TmY9q⊥ → TmY9q⊥
max 1, sum 6
Mi4 → TmY10 → TmY9q⊥
max 3, sum 28
Tm1 → Dm3v → TmY9q⊥
max 0, sum 4
Tm1 → TmY9q⊥ → Dm3q → TmY9q⊥
